# Estimating the Prevalence of Cardiac Amyloidosis in Old Patients with Heart Failure—Barriers and Opportunities for Improvement: The PREVAMIC Study

**DOI:** 10.3390/jcm12062273

**Published:** 2023-03-15

**Authors:** Rocío Ruiz-Hueso, Prado Salamanca-Bautista, Maria Angustias Quesada-Simón, Sergi Yun, Alicia Conde-Martel, José Luis Morales-Rull, Roi Suárez-Gil, José Ángel García-García, Pau Llàcer, Eva María Fonseca-Aizpuru, Beatriz Amores-Arriaga, Ángel Martínez-González, Arola Armengou-Arxe, José Luis Peña-Somovilla, Manuel Lorenzo López-Reboiro, Óscar Aramburu-Bodas

**Affiliations:** 1 Internal Medicine Department, Hospital Universitario Virgen Macarena, Avda. Dr. Fedriani, 3, 41009 Sevilla, Spain; 2 Department of Medicine, Universidad de Sevilla, San Fernando, 4, 41004 Sevilla, Spain; 3 Internal Medicine Department, Hospital Universitario La Paz, Plaza de la Castellana, 261, 28046 Madrid, Spain; 4 Community Heart Failure Program, Cardiology Department, Bellvitge University Hospital, L’Hospitalet de Llobregat, Carrer de la Feixa Llarga, s/n., 08907 Barcelona, Spain; 5 Department of Internal Medicine, Bellvitge University Hospital, L’Hospitalet de Llobregat, 08907 Barcelona, Spain; 6 Bio-Heart Cardiovascular Diseases Research Group, Bellvitge Biomedical Research Institute (IDIBELL), L’Hospitalet de Llobregat, 08907 Barcelona, Spain; 7 Internal Medicine Department, Hospital Universitario Dr. Negrín, Pl. Barranco de la Ballena s/n. 35010 Las Palmas de Gran Canaria, Spain; 8 Internal Medicine Deparment, Hospital Universitario Arnau de Vilanova, IRBLleida, Avda. Alcalde Rovira Roure, 80, 25198 Lérida, Spain; 9 Internal Medicine Department, Hospital Universitario Lucus Augusti, Rua Dr. Ulises Romero, 1, 27003 Lugo, Spain; 10 Internal Medicine Department, Hospital Universitario Virgen del Valme, Ctra. Cádiz, km 548,9, 41014 Sevilla, Spain; 11 Internal Medicine Department, Hospital Universitario Ramón y Cajal, IRYCIS, M-607, 9, 100, 28034 Madrid, Spain; 12 Internal Medicine Department, Hospital de Cabueñes, C/Los Prados, 395, 33394 Gijón, Spain; 13 Internal Medicine Deparment, Hospital Universitario Lozano Blesa, C/San Juan Bosco, 15, 50009 Zaragoza, Spain; 14 Internal Medicine Department, C/Altos de Nava, s/n., 24071 León, Spain; 15 Internal Medicine Department, Leon University Hospital Complex, Hospital Universitario Josep Trueta, Avinguda de Franca s/n., 17007 Gerona, Spain; 16 Internal Medicine Department, Hospital San Pedro., C/Piqueras, 98, 26006 Logroño, Spain; 17 Internal Medicine Department, Hospital Comarcal Monforte de Lemos., Rua Corredoira s/n., 27400 Monforte de Lemos, Spain

**Keywords:** cardiac amyloidosis, epidemiology, heart failure, prevalence

## Abstract

Background: Cardiac amyloidosis (CA) could be a common cause of heart failure (HF). The objective of the study was to estimate the prevalence of CA in patients with HF. Methods: Observational, prospective, and multicenter study involving 30 Spanish hospitals. A total of 453 patients ≥ 65 years with HF and an interventricular septum or posterior wall thickness > 12 mm were included. All patients underwent a ^99m^Tc-DPD/PYP/HMDP scintigraphy and monoclonal bands were studied, following the current criteria for non-invasive diagnosis. In inconclusive cases, biopsies were performed. Results: The vast majority of CA were diagnosed non-invasively. The prevalence was 20.1%. Most of the CA were transthyretin (ATTR-CM, 84.6%), with a minority of cardiac light-chain amyloidosis (AL-CM, 2.2%). The remaining (13.2%) was untyped. The prevalence was significantly higher in men (60.1% vs 39.9%, *p* = 0.019). Of the patients with CA, 26.5% had a left ventricular ejection fraction less than 50%. Conclusions: CA was the cause of HF in one out of five patients and should be screened in the elderly with HF and myocardial thickening, regardless of sex and LVEF. Few transthyretin-gene-sequencing studies were performed in older patients. In many patients, it was not possible to determine the amyloid subtype.

## 1. Introduction

Heart failure is a condition that predominantly affects older patients [1] and is the leading cause of hospitalization in the over-65 age group [2]. In recent years, cardiac amyloidosis (CA), considered a rare disorder, has emerged as an underdiagnosed cause of heart failure [3]. More than 98% of CA is due to transthyretin-associated amyloidosis cardiomyopathy (ATTR-CM), in its hereditary (ATTRv-CM) or wild-type form (ATTRwt-CM), or to primary amyloidosis (AL-CM) [4,5].

Interest in ATTR-CM has been increasing in the last few years due to the development of non-invasive techniques that have facilitated diagnosis and new therapeutic options that prolong life in these patients [6,7]. ATTR-CM has been identified in 13% of patients with HF with a preserved left ventricular ejection fraction [8] and in 6–15% of patients with aortic stenosis [9,10]. However, the studies on the prevalence of CA have heterogeneous designs and they are mostly retrospective or single-centre, so they do not allow us to determine the exact prevalence of this pathology within heart failure.

In addition, there are still many gaps in the knowledge of the disease. Although CA has been mainly associated with males with heart failure with a preserved left ventricular ejection fraction [8,9,10,11], it is not clear whether women are underrepresented in the studies and the proportion of patients with CA who have a reduced/mildly reduced left ventricular ejection fraction. The prevalence in the very old patient is also unknown due to the difficulties in conducting studies, including real-life trials, in this age group.

Patients admitted for heart failure in Spain are mainly cared for by cardiologists and specialists in Internal Medicine. A total of 60% of them, generally elderly, with multiple pathologies and a preserved left ventricular ejection fraction, are seen by specialists in Internal Medicine. In addition, unlike in other countries, there is not a single center to which all patients with amyloidosis are referred. Generally, it is the hospital where they are diagnosed that manages these patients. For these reasons, the *Heart Failure and Atrial Fibrillation Working Group of the Spanish Society of Internal Medicine* decided to carry out a study to estimate the current prevalence of different types of CA in old patients with HF treated in the Internal Medicine setting. 

## 2. Materials and Methods

### 2.1. Study Population

The PREVAMIC was a nationwide, multicentre, observational, cross-sectional, prospective study involving 30 Spanish hospitals (see Appendix A). It started on 1 February 2020, and was closed on 31 March 2021, with a temporary interruption in all centres due to the COVID-19 pandemic (from 15 March 2020, to 31 May 2020). The design of the PREVAMIC study has been published [12]. 

Only those patients of at least 65 years diagnosed with HF according to the *2016 European Society of Cardiology Guidelines* [13] and who had increased left ventricular wall thickness (interventricular septum or posterior wall > 12 mm) were eligible for consideration. Other inclusion criteria were functional *New York Heart Association* class II–IV, diuretic treatment in the last 6 months, and high levels of natriuretic peptides (NT-proBNP > 1800 pg/mL or BNP > 400 pg/mL in acute HF, or NT-proBNP > 600 pg/mL or BNP > 150 pg/mL in a stable situation). The choice of natriuretic peptide cut-off points was made with the idea of ensuring that the patient had HF. For this, the “*European Society of Cardiology Practical Recommendations for the use of peptide concentrations*” were followed [14]. Following the current criteria for non-invasive diagnosis, if the patient agreed to participate, a ^99m^Tc-DPD/PYP/HMDP scintigraphy was performed 1 or 3 h after radiotracer injection using planar imaging. In positive cases by planar scintigraphy, SPECT/CT was subsequently performed to rule out false positives. Blood and urine samples were collected to detect free light-chain abnormalities. In the case of not reaching a diagnosis through the previous tests, cardiac magnetic resonance imaging or cardiac/extracardiac biopsies could be performed. In patients diagnosed with ATTR-CM, transthyretin gene sequencing was strongly recommended. Patients who refused to participate or with active oncological diseases other than myeloma were excluded. Participating hospitals could start recruitment on any date, but they had to invite to participate in the study all inpatients or outpatients who met the inclusion criteria. Patients were included consecutively until reaching the minimum number required for each centre, which was predetermined according to the size of the hospital. Once this number was reached, they could optionally continue recruiting patients, as long as the inclusion was consecutive and all the patients who met the criteria to enter the study were evaluated. 

### 2.2. Study Variables and Data Collection

Data were included in an electronic medical record accessed with a personal password. To preserve confidentiality, no personal data were stored. Age, sex, comorbidities, signs, symptoms, therapies, and the presence of red flags of amyloidosis were included. Laboratory, electrocardiographic, and imaging test data were also included. Other procedures related to the diagnosis of amyloidosis, such as tissue biopsy and genetic testing for ATTR, were also collected. All patients underwent functional and cognitive assessment scales at the time of inclusion in the study. 

### 2.3. Diagnosis of CA 

ATTR-CM was diagnosed by positive findings on ^99m^Tc-DPD/PYP/HMDP scintigraphy (grade 2 or 3 myocardial uptake of radiotracer) and absence of monoclonal protein, with or without confirmation of pathological TTR deposition [6,15]. The diagnosis of ATTRv-CM also required the demonstration of a mutation in the transthyretin gene using a genetic test. For all other types of amyloidosis, it was necessary to demonstrate amyloid deposition in extracardiac or cardiac tissue and findings suggestive of CA on echocardiography or cardiac magnetic resonance imaging. The amyloid subtype was determined in the tissue by immunohistochemistry. 

### 2.4. Ethical Aspects

The patients included were treated following the usual clinical practice. The study was carried out in accordance with the Declaration of Helsinki, and with the Organic Law, on the Protection of Personal Data and Guarantee of Digital Rights. This study was approved by the Clinical Research Ethics Committee of the Virgen Macarena Hospital of Seville (Spain) and is registered on the website ClinicalTrials.gov with the number NCT04066452 (accessed on 10 March 2023). Informed consent was obtained from all participating subjects.

### 2.5. Statistical Analysis

Continuous variables were expressed as mean (95% confidence interval) or median (with 25th to 75th interquartile range), depending on the normality of their distribution. Categorical variables were expressed as frequencies and percentages. A descriptive analysis of the data was carried out, calculating prevalence rates, and a comparative analysis with different variables, such as gender and age. Continuous variables were compared using Student’s t-test or non-parametric Mann–Whitney U-test. Categorical variables were compared using the Chi-square or Fisher’s exact test. Statistical significance was considered as *p* < 0.05. All analyses were performed with the IBM Corp. Released 2015, SPSS Statistics for Windows, Version 23.0. Armonk, NY, USA: IBM Corp.

## 3. Results

A total of 30 hospitals participated in the study. The hospitals had different levels of care. As shown in the study flowchart (Supplementary Material Figure S1), 569 patients were consecutively invited to participate. However, 85 patients did not sign the informed consent document (42 men and 43 women, with a median age of 83 years [79–88]). Thirty-one patients withdrew their consent or died before the tests were performed. Therefore, 453 patients (229 women and 224 men, with a median age 85 years [79–88]) were included. The radiotracer used mostly in the scintigraphy was ^99^Tc-DPD (381, 84%), followed by HMDP (70, 15%) and PYP (2, 1%). 

**Figure 1:**
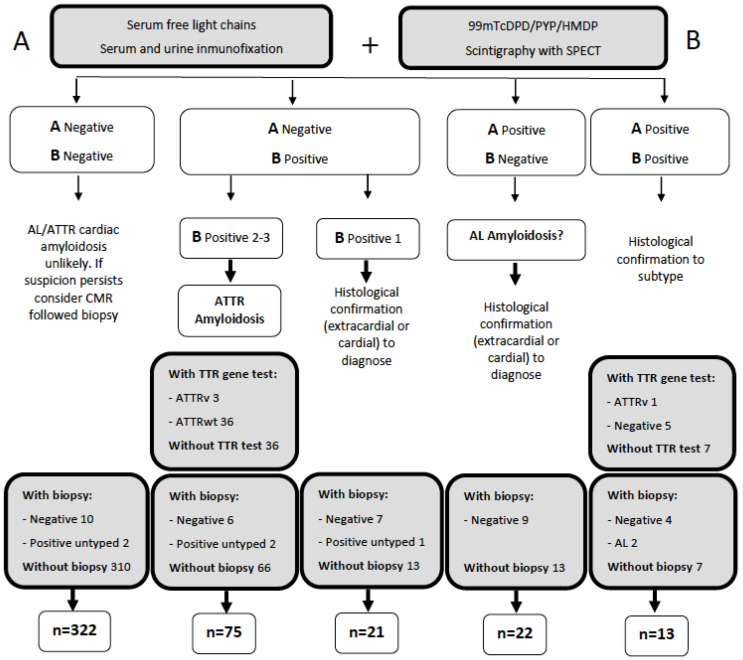
Diagnostic algorithm for cardiac amyloidosis.

As shown in Figure 1, the diagnosis of the vast majority of cardiac amyloidosis was made non-invasively. Specifically, non-invasive methods confirmed the diagnosis of CA in 86 patients; 76 cases were ATTR-CM and 10 cases were untyped. Invasive diagnosis (by four extracardial and one endomyocardial biopsies) was performed in five patients: two AL-CM and three untyped CA. No patient was diagnosed exclusively by nuclear magnetic resonance. Even in the presence of radiological data suggestive of cardiac amyloidosis, a biopsy showing an amyloid was required. For more detailed information about the biopsies and nuclear magnetic resonances performed and their results, see Tables S2 and S3 (Supplementary Material). 

### 3.1. Prevalence of CA in the Heart Failure Cohort 

**Figure 2:**
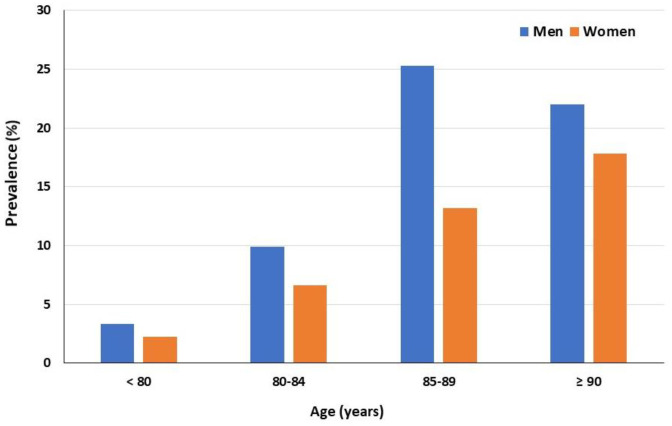
Cardiac amyloidosis prevalence according to age and sex.

Ninety-one patients were diagnosed during the study, and consequently, the prevalence was 20.1%. Of the CA group, 76 cases were ATTR-CM (84.6%), with only two cases of AL-CM (2.2%). In the remaining 13 patients (13.2%), it was not possible to determine if it was ATTR-CM or AL-CM. At least 5.2% of patients with ATTR-CM had a hereditary cause, but there was a significant proportion of patients who did not undergo genetic testing. The prevalence was significantly higher in men (60.1% vs 39.9%, *p* 0.019) and it increased progressively with age (Figure 2). Although HFpEF predominated, 26.5% of patients with CA had a left ventricular ejection fraction <50%. 

### 3.2. Characteristics of the Patients with and without CA

The clinical characteristics and the treatment of the patients with and without CA are detailed in Table 1. Patients with CA were significantly older, more often male, and had more fatigue and muscle weakness than those without the disease. In terms of red flags, patients with CA more frequently had carpal tunnel syndrome, lumbar spinal stenosis, biceps tendon rupture, and monoclonal gammopathy of uncertain significance. Intolerance to angiotensin-converting enzyme inhibitors, angiotensin receptor blockers, and beta-blockers was also more common in the group of patients with CA. 

The differences between the electrocardiogram and echocardiography are summarized in Table 2. CA patients more frequently had a low voltage on the electrocardiogram. The most suggestive findings of CA on the echocardiography were the presence of pericardial effusion and speckled myocardium, together with a thicker interventricular septum and posterior wall. Mild aortic stenosis was significantly more frequent in the group of patients with CA, but severe cases were more common in the group of patients without CA.

## 4. Discussion

To the best of our knowledge, this is the first prospective, multicenter study evaluating the prevalence of CA in old patients with heart failure. Amyloidosis was the cause of heart failure in one of five patients with increased left ventricular wall thickness. Although the most common profile of patients with CA was male with heart failure with a preserved ejection fraction, there was a significant proportion of patients with the disease who were women or had HF with a reduced/mildly reduced left ventricular ejection fraction. However, the etiological diagnosis of amyloidosis (typing amyloid in tissue if the non-invasive diagnosis was not conclusive, and genetic testing in ATTR-CM) was achieved in less than half of the patients with CA. 

### 4.1. Prevalence of Cardiac Amyloidosis. Influence of Age, Sex and Left Ventricular Ejection Fraction

Recently, studies on CA, especially on ATTR-CM, have increased enormously. Table S1 (Supplementary Material) details the studies aimed at clarifying its prevalence in patients with heart failure [8,16,17,18,19,20,21,22], mostly single-centre and with a limited sample. The prevalence ranges between 4–20% [8,16,17,18,19,20,21,22]. Our prevalence was the highest, together with that obtained by Lindmark et al. [17], although they included patients with an interventricular septum > 14 mm (vs. > 12 mm in the present study). The inclusion of other types of CA beyond ATTR-CM and very old patients could justify the high prevalence in our study. On the other hand, if we take into account the patients who refused to participate in the study and those who died before the tests were performed, the prevalence of CA could have been lower (16%).

Another relevant finding was the proportion of women with CA (Figure 2), the highest in the series published to date [11,23,24]. For unclear reasons, CA has traditionally been more reported in men. Several factors have been proposed to explain this fact, including the cardioprotective effects of estrogens, diagnostic challenges in women, or lack of female representation in cohorts. The prevalence of women with CA in systematic reviews and meta-analyses is 9–13% [11,23,24], increasing to 30% in a recent retrospective study conducted in Sweden [25]. A similar number of men and women were included in our cohort and 40% of patients affected with CA were women. Therefore, a lower presence of women in other studies could justify our data. Moreover, most of the studies conducted to date have focused on heart failure with a preserved ejection fraction, but 26.5% of our CA patients had a left ventricular ejection fraction < 50%. Although data are limited, a study that analyzed positive scintigraphies for TTR deposits showed that 31% of them had reduced LVEF [26]. Consistent with our data, a Spanish cohort of patients with CA has shown that up to a third of patients may have heart failure with a reduced left ventricular ejection fraction [27], reaching even 50% in other studies that include older patients [17]. In our study, almost 30% of the patients with CA had ischemic heart disease, so the reduced LVEF could be related to this fact, in addition to progressive CA. However, diagnostic suspicion should not focus exclusively on the classic profile of men with heart failure a with preserved left ventricular ejection fraction, since a significant number of patients with the disease could go unnoticed. Among the patients diagnosed with CA, a large proportion were diagnosed during a hospital admission, so it is important to emphasize that this disease should be suspected in patients with a poor evolution of their heart failure and recurrent readmissions [5].

### 4.2. Barriers and Opportunities for Improvement

The diagnostic process and therapeutic approach of the old patients have certain singularities. It is fundamental to keep a balance between the procedures necessary to reach a correct diagnosis and the benefit that we can obtain from them, always respecting the preferences of the patient. CA is a pathology that could improve with specific treatments although there are no cost-effectiveness studies in the geriatric population. Furthermore, it is managed differently from other heart failure etiologies due to its intolerance to drugs, predisposition to atrio-ventricular blocks requiring pacemakers, and high rates of atrial fibrillation [5]. However, CA continues to be underdiagnosed despite its prevalence, which is very remarkable and increases enormously after the age of 85 years. It is therefore necessary to increase awareness of this disease among physicians who care for patients with heart failure.

However, even in the cases identified as CA in our study, there were significant difficulties in reaching the etiological diagnosis of the amyloidosis subtype, even though the treatment and prognosis of AL-CM and ATTR-CM are completely different. In many of them, no further explorations were performed due to the wishes of the patients, poor accessibility to genetic tests in some centres, difficulty in providing a disease-modifying treatment, and limited access to specialists. The low number of genetic tests performed in patients with ATTR-CM in our study is especially striking and it shows the lack of knowledge about the disease outside of the specialist centres. This test, minimally invasive, should be performed routinely on all diagnosed patients since there are transthyretin gene mutations that present as late-onset cardiomyopathy [28]. In addition, a family screening should be done in affected patients. 

Despite these difficulties, the management of cardiac amyloidosis has many opportunities to improve. Many advances are being made towards an earlier diagnosis that allows for more effective treatment. Speckle-tracking echocardiographic imaging techniques are playing a role in diagnosis in less advanced stages. In addition, left atrial dysfunction is emerging as an early marker of amyloid heart disease and a predictor of complications [29,30]. Similarly, other recently developed tools such as scores performed in heart failure with a preserved left ventricular ejection fraction or machine-learning models that identify patients at a high risk of amyloidosis could be of benefit to our patients [31,32,33]. Lastly, there are clinical pathways implemented in other countries with excellent results [34,35,36]. These programs, focused on multidisciplinary collaboration, have shown increased awareness of the disease among physicians, improvement in diagnostic delay, and better access to amyloid specialists.

### 4.3. Strengths and Limitations

The main strength of the study is its prospective and multi-centre design. The cohort is contemporary, and representative of real-life patients with heart failure treated in Internal Medicine departments. A careful evaluation was performed to avoid bias. However, the study also has some limitations. First, the inclusion of older patients has not allowed us to determine the type of amyloid in a remarkable proportion of CA. Similarly, a large part of ATTR-CM has not undergone a genetic study. The lack of information about genetic testing affects the quality of the epidemiological findings since it prevents us from knowing the exact prevalence of ATTRwt-CM and ATTRv-CM. Furthermore, the tests carried out in patients that could not be diagnosed using a non-invasive method were not standardized in all patients. Finally, almost 20% of the initially screened patients declined the invitation to participate. Although the median age and the proportion of men and women were similar to those who participated in the study, it could have selected the patients and altered the prevalence of CA. 

## 5. Conclusions

CA, especially ATTRwt-CM, is a common, under-diagnosed, and age-related cause of HF. It should be suspected in old patients with heart failure and increased left ventricular wall thickness, regardless of the patient’s sex or left ventricular ejection fraction, even if the patient already has a known heart disease. In this environment, ATTR-CM represents the most prevalent amyloidosis with cardiac involvement, with a much higher presence than AL-CM. The etiological study of the amyloid subtype in old patients remains incomplete in many of them. It is necessary to establish strategies that allow an early diagnosis and an improvement in the management of patients with CA.

## Figures and Tables

**Table 1:** Baseline characteristics of the population.

	Cardiac Amyloidosis n = 91 (20.1%)	No Cardiac Amyloidosis n = 372 (79.9%)	All n = 453 (100%)	*p* Value
Demography/physical examination Age, median (IQR), years Women, n (%) BMI, median (IQR) Kg/m^2^ SBP, median (IQR), mmHg Outpatients	88 [85–91] 36 (39.9) 27.1 [25.0–32.7]	83 [78–87] 193 (53.5) 30.1 [26.6–33.9]	85 [79–88] 229 (50.6) 29.8 [26.1–32.7]	<0.001 0.019 0.002
	126 [109–140] 50 (54.9)	129 [116–142] 263 (70.1)	128 [115–142] 313 (69.0)	0.442 0.003
Heart disease/Devices Coronary artery disease, n (%) Moderate/severe valve disease, n (%) Previous HF, n (%) Pacemaker, n (%)	27 (29.7) 42 (39.6) 77 (84.6) 19 (20.9)	112 (30.9) 189 (48.0) 321 (88.7) 53 (14.7)	139 (30.7) 231 (46.4) 398 (87.9) 72 (15.9)	0.411 0.253 0.397 0.125
Other comorbidities Diabetes mellitus, n (%) Hypertension, n (%) Dyslipidemia, n (%) Cerebrovascular disease, n (%) COPD, n (%)	27 (29.7) 78 (85.7) 50 (54.9) 11 (12.1) 9 (9.9)	151 (41.7) 321 (88.7) 211 (58.3) 69 (19.1) 69 (19.1)	178 (39.3) 399 (88.1) 261 (57.6) 80 (17.7) 78 (17.2)	0.137 0.732 0.810 0.295 0.103
Red flags Low BP, previous hypertension, n (%) History of syncope, n (%) History of back pain, n (%) History of anaemia, n (%) Intolerance of ACEI/ARBs, n (%) Intolerance of beta-blockers, n (%) Intolerance of CCBs, n (%) Intolerance of digoxin, n (%) Carpal tunnel syndrome, n (%) Spinal stenosis, n (%) Biceps tendon ruptura, n (%) Periorbital purpura, n (%) Macroglosia, n (%) MGUS, n (%) Peripheral neuropathy, n (%) Autonomic dysfuncion, n (%) Nephrotic syndrome, n (%)	22 (24.2) 7 (7.7) 31 (34.1) 41 (45.1) 8 (8.8) 15 (16.5) 2 (2.2) 4 (4.4) 21 (23.1) 8 (8.8) 3 (3.3) 1 (1.1) 2 (2.2) 11 (12.1) 11 (12.1) 2 (2.2) 0 (0)	48 (13.3) 36 (9.9) 119 (32.9) 170 (47.0) 12 (3.3) 23(6.4) 5 (1.4) 8 (2.2) 17 (4.7) 19 (5.2) 1 (0.3) 1 (0.3) 1 (0.3) 16 (4.4) 20 (5.5) 8 (2.2) 6 (1.7)	70 (15.5) 43 (9.5) 150 (33.1) 211 (46.6) 20 (4.4) 38 (8.4) 7 (1.5) 12 (2.6) 38 (8.4) 27 (6.0) 4 (0.9) 2 (0.4) 3 (0.7) 27 (6.0) 31 (6.8) 10 (2.2) 6 (1.3)	0.033 0.564 0.077 0.811 0.014 0.009 0.116 0.578 <0.001 0.001 0.012 0.772 0.221 0.047 0.068 0.980 0.478
Signs and symptoms Dyspnoea, n (%) Fatigue, n (%) Muscular weakness, n (%) Eye symptoms, n (%) Dry cough, n (%) Angina, n (%) Palpitations, n (%) Weightloss, n (%) Diarrhea, n (%) Constipation, n (%) Paresthesia, n (%) Delusionsm n (%)	67 (73.6) 69 (75.8) 44 (48.4) 15 (16.5) 14 (15.4) 10 (11) 13 (14.3) 17 (18.7) 9 (9.9) 17 (18.7) 12 (13.2) 8 (8.8)	260 (71.8) 196 (54.1) 108 (29.3) 68 (18.8) 55 (15.2) 30 (8.3) 80 (22.1) 36 (9.9) 21 (5.8) 82 (22.7) 27 (7.5) 16 (4.4)	327 (72.2) 265 (58.5) 152 (33.6) 83 (18.3) 69 (15.2) 40 (8.8) 93 (20.5) 53 (11.7) 30 (6.6) 99 (21.9) 39 88.6) 24 (5.3)	0.573 0.001 0.002 0.866 0.992 0.487 0.248 0.067 0.363 0.715 0.214 0.227
Functional assessment Previous NYHA class III-IV, n (%) Barthel Index, median (IQR), points Pfeiffer Questionnaire, errors	27 (31.0) 90 [70–100] 1 [0–2]	79 (22.8) 90 [80–100] 1 [0–2]	106 (24.4) 90 [75–100] 1 [0–2]	0.108 0.237 0.575
Laboratory Haemoglobin, median (IQR), mg/dl Creatinine, median (IQR), mg/dl Sodium, median (IQR), mEq/L Potassium, median (IQR), mEq/L Troponin T-hs, median (IQR), ng/L NT-proBNP, median (IQR), pg/mL Ca125, median (IQR), U/mL	12.8 [11.5–13.7] 1.3 [1.0–1.8] 141 [137–143] 4.4 [4.1–4.9] 81.7 [40.2–132.5] 4398 [2035–8452] 33.1 [12.7–94.4]	12.4 [11.0–13.8] 1.3 [0.9–1.7] 141 [139–143] 4.3 [4.0–4.8] 32.2 [22.0–58.0] 2420 [1368–4352] 17.7 [10.5–35.7]	12.4 [11.1–13.8] 1.3 [1.0–1.7] 141 [139–143] 4.4 [4.0–4.8] 38.0 [24.0–72.0] 2651 [1498–5271] 18.5 [11.0–39.5]	0.206 0.828 0.182 0.194 <0.001 0.001 0.065
Treatment Beta-blockers, n (%) ACEIs, n (%) ARBs, n (%) Aldosterone antagonists, n (%) Sacubitril-valsartan, n (%) Digoxin, n (%) CCBs, n (%) Nitrates, n (%) Loop diuretics, n (%) Thiazide diuretics, n (%) i-SGLT2s, n (%) Anticoagulants, n (%)	46 (50.5) 23 (25.3) 23 (25.3) 25 (27.2) 6 (6.6) 3 (3.3) 14 (15.4) 7 (7.7) 77 (84.6) 13 (14.3) 1 (1.1) 59 (64.8)	249 (68.8) 102 (28.2) 112 (30.9) 99 (27.3) 34 (9.4) 29 (8.0) 104 (28.7) 50 (13.8) 328 (90.6) 51 (14.1) 27 (7.5) 234 (62.9)	295 (65.1) 125 (27.6) 135 (29.8) 124 (27.4) 40 (8.8) 32 (7.1) 118 (26.0) 57 (12.6) 405 (89.4) 64 (14.1) 28 (6.2) 293 (64.7)	0.004 0.852 0.572 0.756 0.695 0.209 0.034 0.286 0.209 0.948 0.077 0.959

ACEI: angiotensin-converting enzyme inhibitor; ARB: angiotensin II receptor blockers, BMI: body mass index; BP: blood pressure; CCB: calcium channel blocker; COPD: chronic obstructive pulmonary disease; ICD: implantable cardioverter defibrillator; IQR: interquartile range; i-SGLT2: sodium-glucose-linked cotransporter-2 inhibitors; MGUS: monoclonal gammopathy of uncertain significance; NYHA: New York Heart Association; SBP: systolic blood pressure.

**Table 2:** Electrocardiogram and echocardiogram parameters.

	Cardiac Amyloidosis n = 91 (20.1%)	No Cardiac Amyloidosis n = 372 (79.9%)	All n = 453 (100%)	*p* Value
Electrocardiogram Atrio-ventricular block Atrial fibrillation Low voltage Pseudo-myocardial infarction pattern Right bundle branch block Left bundle branch block Left ventricular hypertrophy	9 (9.9) 66 (72.5) 28 (30.8) 16 (17.6) 25 (27.5) 19 (20.9) 19 (20.9)	37 (10.0) 250 (69.1) 59 (16.3) 65 (18.0) 92 (25.4) 98 (27.1) 121 (33.4	46 (10.1) 316 (69.8) 87 (19.2) 81 (17.9) 117 (25.8) 117 (25.8) 140 (30.9)	0.918 0.687 0.004 0.361 0.774 0.458 0.047
Echocardiogram LVEF < 40% LVEF 40–49% LVEF > 50% SWT, median (IQR), mm PW, median (IQR), mm LV mass index (IQR), g/m^2^ Left atrium (mm) Aortic stenosis Aortic regurgitation Mitral stenosis Mitral regurgitation Tricuspid regurgitation TAPSE, median (IQR), mm Pericardial effusion Speckled myocardium	8 (9.6) 14 (16.9) 61 (73.5) 17 [14–19] 14 [13–17] 151 [125–187] 47 [43–51] 16 (20.1) 40 (52.6) 2 (2.5) 61 (78.2) 56 (71.8) 18 [16–23] 22 (24.2) 16 (17.6)	42 (12.1) 37 (10.6) 259 (77.3) 14 [13–15] 13 [12–14] 135 [107–171] 46 [42–50] 75 (22.2) 152 (45.4) 26 (7.7) 265 (78.2) 220 (65.7) 20 [17–25] 37 (10.2) 8 (2.2)	50 (11.6) 51 (11.8) 330 (76.6) 14 [13–16] 13 [12–14] 138 [112–174] 47 [43–52] 91 (21.9) 192 (46.7) 28 (6.7) 326 (78.2) 276 (66.8) 20 [17–25] 59 (13.0) 24 (5.3)	0.263 <0.001 <0.001 0.056 0.325 0.030 0.066 0.187 0.157 0.104 0.290 <0.001 <0.001

IQR: interquartile range; LV: left ventricular; LVEF: left ventricular ejection fraction; PS: posterior wall; SWT: septal wall thickness; TAPSE: tricuspid annular plane systolic excursion.

## Data Availability

The data are contained within the article.

## Supplementary Materials

The following supporting information can be downloaded at: https://www.mdpi.com/article/10.3390/jcm12062273/s1, Figure S1: Study flowchart; Table S1: Prevalence studies evaluating amyloidosis as a cause of heart failure, Table S2: Biopsies performed: location and result, Table S3: Cardiac magnetic resonance imaging.

## Appendix A

### PREVAMIC Study Researchers

Amores-Arriaga B (Hospital Universitario Lozano Blesa, Zaragoza), Amorós-Martínez F (Hospital Vinalopó, Elche), Andrés-Imaz N (Hospital de Mendaro, Mendaro), Aramburu-Bodas O (Hospital Universitario Virgen Macarena, Sevilla), Armengou-Arxe A (Hospital Josep Trueta, Gerona), Bermudo-Guitarte C (Hospital Universitario Virgen Macarena, Sevilla), Bernardo-Galán P (Hospital de Mendaro, Mendaro), Bonache-Bernal F (Hospital de Mendaro, Mendaro), Calero-Molina E (Hospital Universitario de Bellvitge, L’Hospitalet de Llobregat), Calvo-Morón MC (Hospital Universitario Virgen Macarena, Sevilla), Casado-Cerrada J (Hospital Universitario de Getafe, Getafe), Castillo-Paredes M (Hospital Juan Ramón Jiménez, Huelva), Cepeda-Rodrigo JM (Hospital de la Vega Baja, Orihuela), Choucino-Fernández T (Hospital Universitario A Coruña, La Coruña), Conde-Martel A (Hospital Universitario Dr. Negrín, Las Palmas de Gran Canaria), Cuadrat-Begue I (Hospital de Santa María, Lérida), Delgado-Verges C (Hospital de Cabueñes, Gijón), Díez-Manglano J (Hospital Royo Villanova, Zaragoza), Domingo-Baldrich E (Hospital Vall D’Hebron, Barcelona), Fernández-Soler C, Fiteni-Mera I (Hospital Royo Villanova, Zaragoza), Fonseca-Aizpuru EM (Hospital de Cabueñes, Gijón), Formiga F (Hospital Universitario de Bellvitge, L’Hospitalet de Llobregat), García-García JA (Hospital Universitario Virgen del Valme, Sevilla), García-Fernández-Bravo I (Hospital Universitario Gregorio Marañón, Madrid), González-Moraleja J (Hospital Virgen de la Salud, Toledo), Liroa-Romero MF (Hospital Universitario Lucus Augusti, Lugo), Llàcer-Iborra P (Hospital Universitario Ramón y Cajal, Madrid), López-Reboiro ML (Hospital Comarcal Monforte de Lemos, Lugo), Martínez-González A (Hospital de León, León), Martínez-Rodés P (Hospital Royo Villanova, Zaragoza), Méndez-Bailón M (Hospital Clínico Universitario San Carlos, Madrid), Montero-Hernández E (Hospital Universitario Puerta del Hierro, Majadahonda), Morales-Rull JL (Hospital Universitario Arnau de Vilanova, Lérida), Moya-Saiz MJ (Hospital Virgen de la Salud, Toledo), Muela-Molinero A (Hospital de León, León), Navarro-Sáez MC (Hospital Parc Taulí, Sabadel), Pacheco-Castellanos MC (Hospital Vinalopó, Elche), Páez-Rubio I (Hospital Juan Ramón Jiménez, Huelva), Peña-Somovilla JL (Hospital de San Pedro, Logroño), Pérez-Bocanegra C (Hospital Vall D’Hebron, Barcelona), Quesada-Simón MA (Hospital Universitario La Paz, Madrid), Redondo-Galán MP (Hospital Virgen de la Salud, Toledo), Ruiz-Hueso R (Hospital Universitario Virgen Macarena, Sevilla), Salamanca-Bautista P (Hospital Universitario Virgen Macarena, Sevilla), Sánchez-Castaño AJ (Hospital Virgen de la Salud, Toledo), Seoane-González B (Hospital Universitario A Coruña, La Coruña), Soler-Rangel ML (Hospital Infanta Sofía, San Sebastian de los Reyes), Soriano-Sánchez T (Hospital Vall D’Hebron, Barcelona), Suárez-Gil R (Hospital Universitario Lucus Augusti, Lugo), Vaquero-Hernández JM (Hospital de Mendaro, Mendaro), Vicente-Rodrigo JA (Hospital de Sagunto, Sagunto), Yun S (Hospital Universitario de Bellvitge, L’Hospitalet de Llobregat).
