# Supplementary material for: Estimating the Prevalence of Cardiac Amyloidosis in Old Patients with Heart Failure—Barriers and Opportunities for Improvement: The PREVAMIC Study
Source: J Clin Med. 2023 Mar 15;12(6):2273. doi: 10.3390/jcm12062273 (PMC10057876; doi:10.3390/jcm12062273)
Supplement: Supplementary file 1 [file jcm-12-02273-s001.zip › jcm-2216577-supplementary.pdf]

Supplementary Materials:

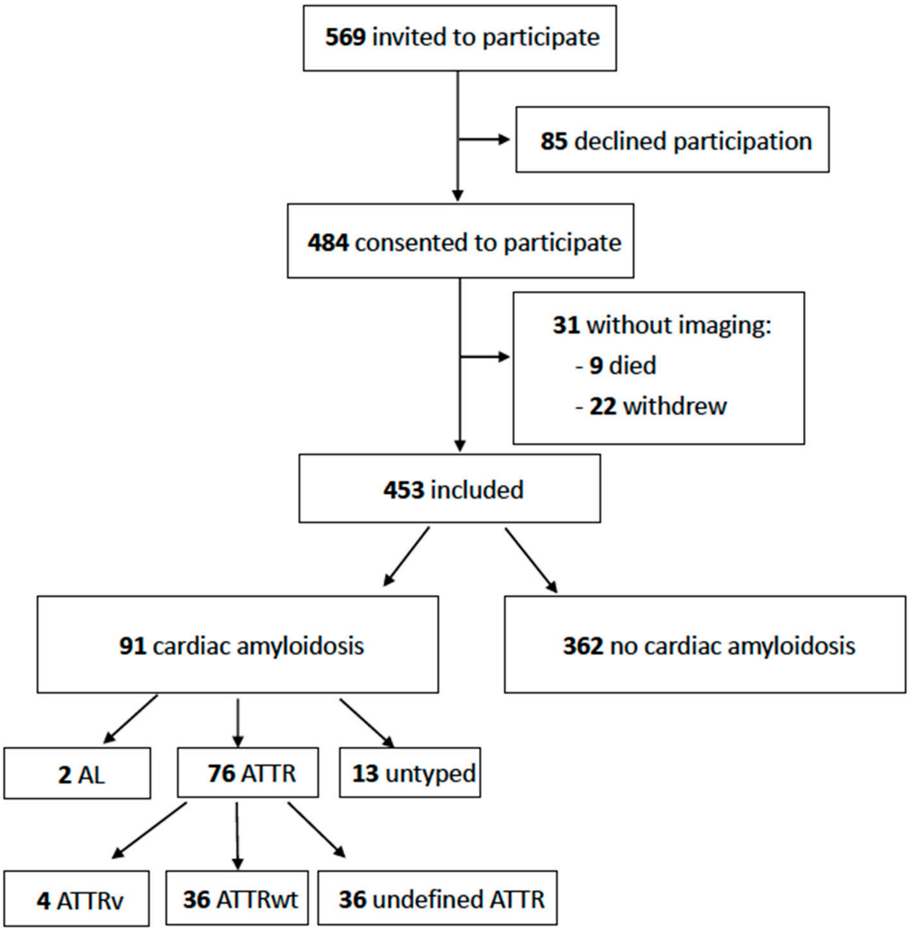

Figure S1: Study flowchart

Table S1: Prevalence studies evaluating amyloidosis as a cause of heart failure

Author (year of publication)	Type of study	Patients (n)	Age	Inclusion criteria	Prevalence	Diagnostic method	Type of amyloid protein studied
González-López (2015)[8]	Prospective Single-centre	120	82 ± 8	≥ 60 years<br>HFpEF<br>Left ventricular thickness ≥ 12 mm	13.3%	^{99m}Tc-DPD	ATTR
Mohammed (2014)[16]	Prospective Single-centre	HFpEF: 109<br>Controls: 131	HFpEF: 76 ± 13<br>Controls: 69 ± 16	Patients with HF and LVEF>40%<br>Control group (without HF)	HFpEF: 17%<br>Control group: 5%	Autopsies	ATTR
Lindmark (2021)[17]	Prospective Single-centre	174	76.5 ± 12. 7	HF with left ventricular thickness >14 mm.	20%	^{99m}Tc-DPD + study of monoclonal protein	ATTR
Hahn (2020)[18]	Prospective Single-centre	108	66 (57-74)	HFpEF<br>(LVEF ≥ 50%)	14%	Endomyocardial biopsy	ATTR, AL and AA
Devesa (2021)[19]	Prospective Single-centre	58	79 (75-85)	≥ 18 years<br>HFpEF (LVEF ≥ 50%)	5.2%	^{99m}Tc-DPD + study of monoclonal protein	ATTR

				Left ventricular thickness <12 mm.			
AbouEzzeddine (2021)[20]	Population-based cohort study	1235 (total) 286 (screening)	Total: 80 (72-87) Screening: 78 (71-84)	≥ 60 years HFpEF (LVEF ≥ 40%) Left ventricular thickness ≥ 12mm Southeastern Minnesota	1,3% (Total) 6,3%(Screening)	Pyrophosphate scan + study of monoclonal protein	ATTR
López-Sainz (2019)[21]	Prospective Single-centre	143	79 ± 9	≥ 60 years<br><br>HF with LVEF < 50% + left ventricular thickness ≥ 12mm or conduction disorders	4%	^{99m}Tc-DPD + study of monoclonal protein	AL and ATTR
Ussitalo (2022)[22]	Retrospective Multi-centre	355	80 ± 6	> 70 years HF + scintigraphy performed for non-cardiac reason	7%	^{99m}Tc-HMDP	ATTR
Ruiz-Hueso (Current study, 2022)	Prospective Multi-centre	453	85 [79-88]	≥ 65 years HF with any LVEF value Left ventricular thickness > 12 mm	20.1%	^{99m}Tc-DPD/PYP/HMDP + study of monoclonal protein Biopsy if required	AL and ATTR

AA: secondary amyloidosis; AL: light chain amyloidosis; ATTR: Transthyretin amyloidosis; HF: Heart failure;

HFpEF: heart failure with preserved ejection fraction, LVEF: left ventricular ejection fraction

Table S2: Biopsies performed: location and result

BIOPSIES	n = 56
Location	
Skin (%)	3 (5.3)
Abdominal fat (%)	36 (64.3)
Bone marrow (%)	7 (12.5)
Rectal mucosa (%)	1 (1.8)
Salivary gland (%)	8 (14.3)
Endomyocardial (%)	1 (1.8)
Result	
Light chain amyloidosis (%)	2 (3.6)
Untyped positive (%)	5 (8.9)
Negative (%)	49 (87.5)

*In some patients, more than one biopsy was performed

Table S3: Cardiac magnetic resonance imaging

CARDIAC MAGNETIC RESONANCE IMAGING	n = 51
Late enhancement	
Absent (%)	47 (92.1)
Subendocardial (%)	3 (5.9)
Transmural (%)	1 (2)
T1 mapping	
Typical of amyloidosis (%)	3 (5.9)
Nonspecific (%)	23 (45.1)
Not performed (%)	25 (49.0)
